# A longitudinal study of sexual activity and influencing factors in breast cancer patients during treatment in the Southwest of China: a trajectory analysis model

**DOI:** 10.1186/s12905-024-03150-8

**Published:** 2024-06-18

**Authors:** Zhang Tian, Zhang Xiaolu, Yang Jing, Wen Min, Liao Jiaqian, Chen Shouli, Wang Yingyin, Deng Xiaoyuan, Liu Xiaoyan, Wang Guorong

**Affiliations:** 1 Nursing Department, Sichuan Clinical Research Center for Cancer, Sichuan Cancer Hospital & Institute, Sichuan Cancer Center, Affiliated Cancer Hospital of University of Electronic Science and Technology of China, Chengdu, Sichuan 610041 China; 2 Department of Orthopedic Surgery, West China Hospital, Sichuan University/West China School of Nursing, Sichuan University, Chengdu, Sichuan 610041 China; 3 Ward 2, Breast Surgery Center, Sichuan Clinical Research Center for Cancer, Sichuan Cancer Hospital & Institute, Sichuan Cancer Center, Affiliated Cancer Hospital of University of Electronic Science and Technology of China, Chengdu, Sichuan 610041 China; 4 School of Nursing, Chengdu University of TCM, Chengdu, Sichuan 610075 China; 5 Nursing Department, West China School of Public Health and West China Fourth Hospital,West China Nursing School, Sichuan University, Chengdu, Sichuan 610041 China

**Keywords:** Breast cancer, Sexual activity, Longitudinal study, Trajectory analysis model

## Abstract

**Purpose:**

The aim of this study was to describe the longitudinal developmental trajectories and its influencing factors of sexual activity in patients with breast cancer during treatment.

**Methods:**

A prospective longitudinal study was conducted, including 225 newly diagnosed breast cancer patients in A tumor specialty three-class hospital in Southwest China. We measured sexual activity at the time of admission and diagnosis (T_0_) and one month (T_1_), three months (T_2_), six months (T_3_), and nine months (T_4_) after diagnosis. A trajectory analysis model (GBTM) was used to explore the changes in sexual activity in breast cancer patients. Multivariate binary logistic regression analysis was used to analyse the factors that affected the classification of sexual activity trajectories.

**Results:**

The ratio of sexual activity abruptly declined from 100% at baseline to 39.1% at T_1_. The percentage of sexual activity was improved, from 51.4% at T_2_ to 63.1% at T_4_. The optimal model was a 2-group trajectory of sexual activity in breast cancer patients,36.6% in the “low activity group” and 63.4% in the “high activity group.” The multivariate binary logistic regression analysis revealed statistically significant and positive correlations between sexual activity and age (*β* = 0.085, OR = 1.089, 95%CI 1.035 ∼ 1.145, *P* = 0.001),libido(*β* = 0.774, OR = 2.168, 95%CI 1.337 ∼ 3.515, *P* = 0.002), vaginal lubrication(*β* = 1.254, OR = 33.503, 95%CI 2.000 ∼ 6.137, *P*<0.001).

**Conclusions:**

Breast cancer patients exhibited varying levels of sexual activity during treatment; higher age was associated with increased sexual activity, which can contribute to the recovery of sexual function. Therefore, it is crucial to provide appropriate guidance on sexual health for younger patients.

**Supplementary Information:**

The online version contains supplementary material available at 10.1186/s12905-024-03150-8.

## Introduction

Breast cancer (BC) exhibits the highest global incidence among all types of cancers and is currently experiencing an upward trend [1]. The incidence of BC ranks first among female malignant tumors in China, exhibiting a younger trend [2]. The American Cancer Society reports that the average age for breast cancer diagnosis is 61 years [3, 4]. However, the average age of Chinese patients with breast cancer is in the 45–55 age range, which is younger than that of Western women [5]. Breasts play a significant role in female sexual characteristics. The disconnect between body appearance (such as hair loss from chemotherapy, like loss of an entire breast and scar after mastectomy) and function can be harmful to overall physical, mental, and social functions [6–8]. Therefore, breast cancer is a significant disease threatening women’s lives and health.

With the continuous advancement of early screening, diagnosis, and treatment technologies, the 5-year observed survival rate of breast cancer patients in China has reached an impressive 80.9% [9]. Enhancing the quality of life for survivors has emerged as a prominent focus within breast cancer treatment, with particular emphasis on optimizing sexual well-being as a vital component of overall quality of life. Breast cancer survivors who receive chemotherapy and hormone therapy experience sexuality problems [10–16], including vaginal dryness, dyspareunia and low libido [17–19]. The sexual side effects that result during diagnosis and treatment cause patients to reduce or even suspend their sexual life, significantly reducing the quality of their sexual life [17, 20]. The aforementioned side effects typically remain stable over a specific duration [21–23], however, they may induce sexual dysfunction in patients and directly impact their overall sexual well-being [24, 25].

The current research on the quality of sexual life of breast cancer patients mainly includes two aspects: sexual activity, that is, whether the patient has a sexual life [19, 26, 27]. The other aspect is the problem [28, 29] and influencing factor in sexual life [30–32].

Studies on sexual activity have demonstrated that breast cancer patients who engage in sexual intercourse exhibit a higher average quality of life score compared to those who abstain from sexual activity [19]. Existing longitudinal studies primarily focus on assessing the incidence of sexual activity at different time points to evaluate patients’ overall quality of life [26, 27]. According to DU Hua’s investigation, 29.9% of patients discontinued engaging in sexual intercourse during treatment, and only 58.6% resumed their sexual lives within six months after treatment [26]. The findings from Nancy E. Avis’ study revealed varying percentages of breast cancer patients participating in sexual activities during an eight-month follow-up period, ranging from 52.4–60.7% [27]. However, these studies predominantly encompassed the entire population of breast cancer patients without exploring the heterogeneity regarding their engagement in sexual activities. Furthermore, breast cancer patients are not asked whether they have had sex in the last month, and if they have had sex, the quality of their sexual life is investigated. Otherwise, this measurement of the quality of sexual function may lead to some potentially measurement bias.

The other aspect is the problem and influencing factor in sexual life. Age and surgical removal of the breast are important factors that can affect a patient’s sexual function [30, 31]. Due to the lack of knowledge about sexual life and the influence of traditional culture [33], Chinese breast cancer patients will voluntarily stop sexual life after the disease. They will not recover for a long time [34]. This may be the result of the conservative perception of sexuality in the traditional Chinese culture [35–37]. The survey of sexual activity conducted by Korean foreign scholars only explored the influencing factors of sexual activity at a particular time. Still, it did not examine the trajectory change of patients [32]. Unlike the general population, the sexual life of breast cancer patients involves more emotional support and maintenance of intimacy, and sexual inactivity may hurt the marital life and overall quality of life of patients, similar to depression [29, 38]. Therefore, research on sexual activity is important to improve the quality of sexual life of breast cancer patients [39, 40]. However, there have been limited longitudinal studies and even fewer with samples exceeding 200 survivors to delineate the trajectory of sexual activity and its influencing factors.

Through a prospective longitudinal study, this research aimed to investigate the overall activity status of breast cancer patients during treatment after diagnosis, with the objective of elucidating the trajectory phenomenon of life. Subsequently, our scientific hypothesis posited that variations exist in the trajectory of sexual activity among breast cancer patients undergoing treatment and explored its influencing factors. The objectives of this study were to (1) comprehend the trajectory of sexual activity in patients; (2) determine whether patients exhibited high or low levels of sexual activity during treatment, and (3) explore factors impacting their level of sexual activity, thereby providing theoretical support for clinical patient’s sexual function rehabilitation and effective individualized health guidance.

## Methods

### Description of the study area

The study was conducted at the largest Tertiary A Oncology Hospital in Southwest China. The data were collected from the second ward of breast cancer, which provides comprehensive treatment for breast cancer including surgery, chemotherapy, and targeted therapy. This ward consists of 60 beds and admits an average of 10–15 newly diagnosed patients per week, which can meet the inclusion criteria for research subject.

The conventional treatment for breast cancer patients in the hospital involves a combination of surgery and chemotherapy. Chemotherapy is typically administered in 6–8 cycles, with each cycle lasting 21 days and requiring a 3-day hospital stay. However, if patients experience leukopenia and there are no available beds, their admission time may be affected. The duration of surgery varies depending on the patient’s condition, whether it is before or during chemotherapy, with an average hospital stay of approximately 2 weeks. The aforementioned statement guarantees the integrity and consistency of the data gathered at various time intervals for this research.

### Research design and participants

A longitudinal prospective observation study was designed in this study. The preliminary research findings of our team [41, 42], which has shown that the sexual health of women with breast cancer focuses on their sexual activities. All participants were recruited between September 2019 and January 2021 from a third class tumor hospital in China, and informed consent was obtained.

#### Inclusion criteria

(1) diagnosed with BC for the first time, over 18 years of age; (2) heterosexual, with a stable sexual partner (living together over six months); (3) The presence of organic pathology in the asexual reproductive organs and regular menstrual cycle; (4) can understand and cooperate with the investigation; (5) voluntarily signed informed consent.

#### Exclusion criteria

1) other cancer diagnoses; 2)psychiatric illness diagnoses.

### Sample size calculation

According to the results of a systematic evaluation of a large sample by Jing Liwei in 2019 [43], the incidence of FSD in breast cancer patients was 73.4%. Therefore, according to the calculation method of sample size in Medical Statistics,

$$N = \frac{{{Z_a}^2 \times pq}}{{{d^2}}}$$

where α is the significance level and set to 0.05, then Z_α_≈2, p is the estimated prevalence, q = 1-p, d is The allowable error in the survey was set at 0.1p, at which time the sample size was calculated to be 133 cases, and to ensure the accuracy of the study results, the sample size was increased by approximately 20% and determined to be 160 cases. The final sample size of this study was 225 cases.

### The detailed process diagram

**Fig. 1:**
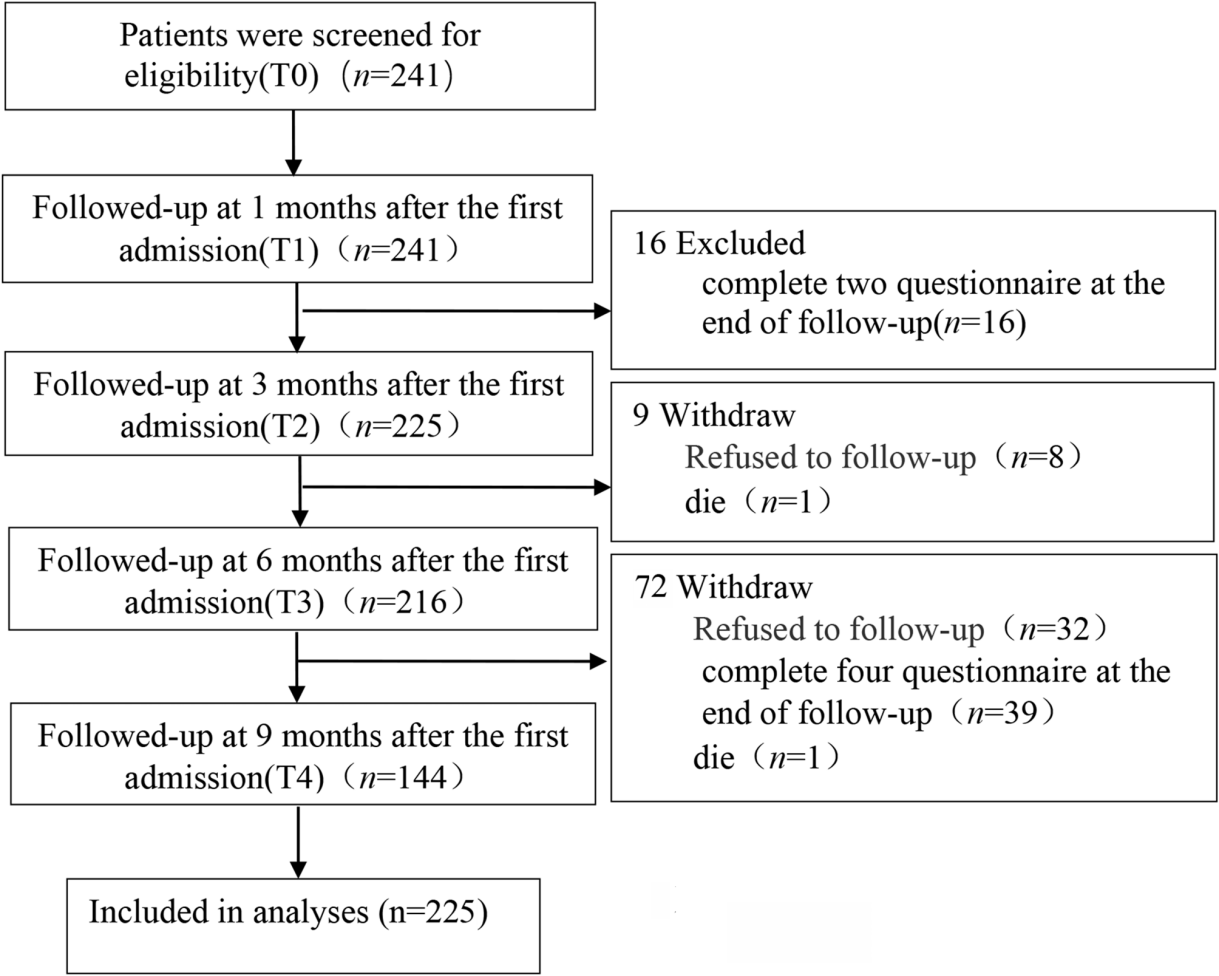
The detailed process diagram

A total of 241 patients were included in this study according to the inclusion and exclusion criteria, and 16 patients who had only 1 follow-up visit by the end of the study were excluded according to the requirements of the GBTM analysis method, resulting in a total valid sample size of 225 patients. A total of 225 patients completed 2 follow-ups, 216 patients completed 3 follow-ups, and 144 patients completed 4 follow-ups, and the detailed enrollment process is shown in Fig. 1.

### Variables

#### Demographic and clinical information sheet

Demographic information (age, place of residence, marital age, occupation, education level, monthly family income, medical insurance) was collected at the time of initial admission; clinical information was ascertained by medical record review at the end of the study.

#### Being sexually active

Women were considered sexually active if they responded “yes” to the question Have you had sex in the past month? Otherwise, women were considered sexually inactive [44].

After extensive discussions among clinical experts, it has been decided to utilize the FSFI as a reliable measure for assessing sexual activity due to the absence of a specialized scale in this domain. However, it is important to acknowledge the limitations of our study during the discussion phase: we solely rely on an initial exploration of this scale to comprehend both the trajectory and influencing factors impacting patients’ sexual activity.

#### Female sexual function index (FSFI)

FSFI is a set of 19 item instrument to measure the sexual function of heterosexual women in the last 4 weeks [45]. Items are designed to measure six domains of sexual health (desire, arousal, lubrication, orgasm, satisfaction, and pain). Total score ranges from 2 to 36. Higher scores indicate better sexual performance. Since there is no classification standard for FSD in China, we refer to the Korean standard [46] and define FSD as below 25 points.

### Ethics consent

The study was conducted in strict accordance with the principles of the Declaration of Helsinki this study was approved by the relevant medical ethics committees (approval numbers: No. 2,016,018). All participants voluntarily participated in the study and could withdraw or retract their data at any time. All the participants received a research information leaflet and signed an informed consent form.

### Data collection

#### Data collection time

After reviewing relevant literature, consulting breast specialists, and considering pre-experimental results, the research team concluded that general demographic data of patients would be collected upon their initial admission. Disease and treatment data would be obtained through the electronic medical record system at the end of the study.

Based on previous literature studies and breast cancer treatment programs, the timing for scale evaluation was determined as follows: first admission, 1 month after diagnosis (approximately 1 cycle of chemotherapy), 3 months after diagnosis (approximately 4 cycles of chemotherapy), 6 months after diagnosis (around hospital discharge), and 9 months after diagnosis (about 3 months post-hospitalization).

#### Data collection methods

Comprehensive case management was implemented with a dedicated nurse providing full nursing care to patients. The nurse conducted one-on-one communication with patients via WeChat. Given that sexual function is considered a private topic, individual visits were arranged in the rehabilitation room of the Breast Department. Patients had limited understanding regarding the orgasmic dimension of female sexual function scale but were well-informed about other dimensions. By thoroughly explaining the scale to patients before completion, we ensured effective quality assurance in filling out the scale.

#### Scale completion time

During hospital treatment, it was decided to fill out the scale on admission day - specifically, on days prior to receiving chemotherapy when patients were in good health. Following hospital treatment completion, specific follow-up times were calculated based on each patient’s initial admission date.

#### Post-discharge follow-up

In the T_4_ follow-up stage, the majority of patients concluded their treatment within the hospital premises, thus necessitating the implementation of WeChat or telephone-based follow-up surveys. During the weChat follow-up process, members of the research team will individually send patients a two-dimensional code of the scale. In cases where patients face difficulties using weChat on their phones or have challenges with reading and comprehension, researchers will contact them via phone to inquire about each item on the scale, promptly recording their responses. If a patient explicitly refuses to continue with the follow-up, it will be discontinued.

The data of this paper comes from our big picture of “Longitudinal study of clinical characteristics and influencing factors of sexual function in breast cancer patients”. All eligible women of the big picture were assessed at five points in time. The data of this paper chose that Baseline questionnaires and the questionnaire of Female sexual function index (FSFI) were completed during the first admission(T_0_). Being sexually active was completed at the first admission(T_0_), at one month (T_1_), at three months (T_2_), at six months (T_3_), and at nine months (T_4_) in the follow-up surveys. To ensure the authenticity of questions about sexual life, the study site chose the rehabilitation room to have a one-on-one conversation with patients and complete the questionnaire.

### Statistical analyses

The data analysis logic in this study is as follows: Firstly, the trajectory analysis of sexual activity was conducted using survey data from clinical studies to comprehend the changes in patients’ sexual activity trajectories. A Group-based Trajectory Model (GBTM) was employed to perform heterogeneous trajectory grouping for sexually active cases at multiple time points, resulting in the identification of high active and low active groups. Finally, univariate and binary logistic regression analyses were performed on the hyperactive and hypoactive groups to explore factors influencing patients’ sexual activity.

### Trajectories of sexual activity

The trajectory analysis was performed using the Proc Traj procedure in SAS 9.4 software to model the sexual activity of breast cancer patients by the Group-based Trajectory Model (GBTM) and fit the optimal trajectory model [47]. The group-based trajectory model (GBTM) was used to identify the trajectories of change in patients’ sexual activity status (active versus inactive). The BTM was conducted using SAS 9.4. BTM is mainly used to analyse longitudinal data in the presence of heterogeneity overall through Proc Traj. The GBTM is mainly used to analyse longitudinal data with heterogeneity in the aggregate and is implemented through the Proc Traj macro in SAS software.

In this study, the sexual activity of breast cancer patients was a dichotomous variable, and a logistic (logit) model was fitted. According to the best trajectory model-fitting method, the optimal number of subgroups and trajectories were selected based on three indicators in order from higher to lower order: (i) Bayesian information criterion (BIC), the closer the BIC value is to 0, the better the model fit is. The average posterior probability of grouping (Average posterior probability, AvePP), which reflects the posterior probability of each individual into the corresponding subgroup after dividing the subgroups according to the trajectory analysis model, is usually taken as an acceptable criterion with AvePP > 0.7. The app values of the nine trajectories in this study are all > 0.7, suggesting that the resultant trajectories fit well. The value of the log Bayes factor (log Bayes factor), which is approximately equal to twice the difference between the BIC of the two compared models, is accepted as a complex model when the value is greater than 6, indicating that the difference in the fitting effect of the two models is large; when the value is less than 2, the difference between the two models is small, and a simple model is used.

### Analysis

To analyse factors affecting the grouping of patients’ sexually active trajectories, a parametric test (t test) was used for information conforming to a normal distribution, nonparametric tests (χ^2^ test, Mann–Whitney U test, Kruskal–Wallis H test) were used for categorical variables or nonnormal distribution, and binary logistic regression analysis was used for multifactor analysis. In this study, we incorporated age as a variable in the multifactorial analysis, following the Chinese breast cancer screening guidelines [48], to investigate its impact on the sexual activity of breast cancer patients.

## Results

**Fig. 2:**
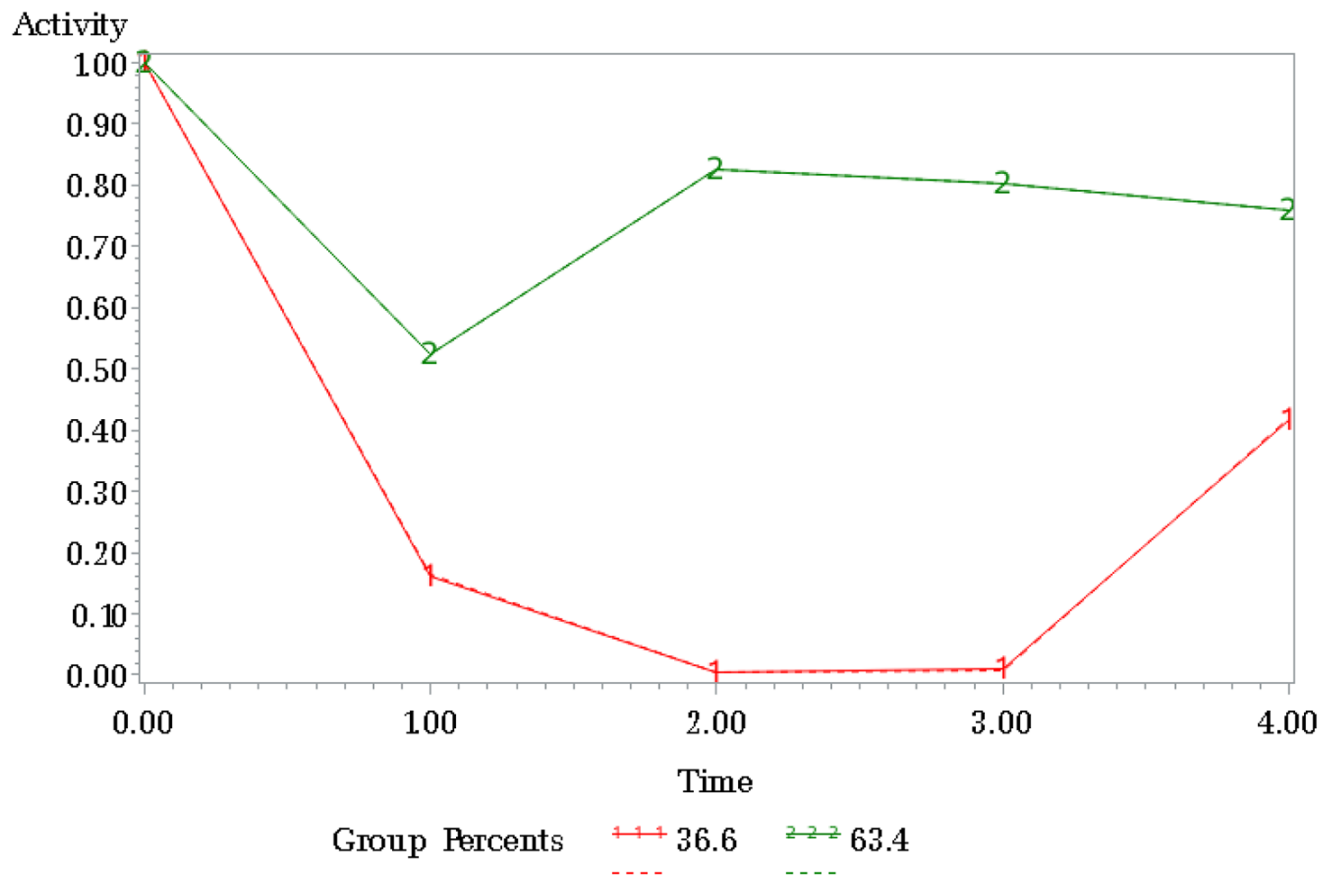
Trajectory of sexual activity from 1 month to 9 months after diagnosis (subgroup 1: low-activity group; subgroup 2: high-activity group)

Corresponding to the principles of data analysis, the findings of this study were presented as follows: Firstly, an overview of the demographic characteristics of 225 patients was provided, with specific data displayed in Table 1. Subsequently, the overall proportion of sexual activity from T0 to T4 among these patients was reported in Table 2. The Group Based Trajectory Model (GBTM) was employed to categorize diverse trajectories for sexually active cases at multiple time points, resulting in the identification of high active and low active groups, with specific data displayed in Table 3; Fig. 2. Lastly, univariate and binary logistic regression analyses(see Tables 1 and 4) were conducted within both hyperactive and hypoactive groups to investigate factors influencing patients’ sexual activity, with specific data displayed.

**Table 1:** Comparison of demographic characteristics of different sexually active trajectory subgroups(*n* = 225)

Variables	Total(*n* = 225)	Low-activity group (*n* = 87)	High-activity group (*n* = 138)	Statistical value	*P*
**Residence**					
Town	152(67.56%)	55(63.20%)	97(70.30%)	1.217^b^	0.270
Country	73(32.44%)	32(36.80%)	41(29.70%)		
**Occupation**					
Cadres and professional and technical personnel	65(28.89%)	28(32.20%)	37(26.80%)	2.037 ^b^	0.729
Worker	48(21.33%)	15(17.20%)	33(23.90%)		
Farmer	37(16.44%)	16(18.40%)	21(15.20%)		
Retiree	29(12.89%)	11(12.60%)	18(13.00%)		
Non-employment	46(20.45%)	17(19.50%)	29(21.00%)		
**Literacy**					
Primary school	50(22.22%)	22(25.30%)	28(20.30%)	4.003^c^	0.261
Middle school	89(39.56%)	31(35.6%)	58(42.0%)		
High school or technical secondary school	45(20.00%)	14(16.10%)	31(22.50%)		
College degree or above	41(18.22%)	20(23.00%)	21(15.20%)		
**Household income level**					
<2000	91(40.44%)	32(36.80%)	59(42.80%)	1.030^c^	0.598
2000–5000	98(43.56%)	39(44.80%)	59(42.80%)		
>5000	36(16.00%)	16(18.40%)	20(14.50%)		
**Medical insurance classification**					
Urban medical insurance	93(41.33%)	33(37.90%)	60(43.50%)	2.105^b^	0.551
New farming	74(32.89%)	27(31.00%)	47(34.10%)		
Commercial insurance	25(11.11%)	12(13.80%)	13(9.40%)		
Other insurance	33(14.67%)	15(17.20%)	18(13.00%)		
**Menstrual states**					
Menopause	76(33.78%)	30(34.50%)	46(33.30%)	0.032^b^	0.859
Non-menopause	149(62.22%)	57(65.50%)	92(66.70%)		
**Underlying disease**					
No basic disease	181(80.44%)	67(77.00%)	114(82.60%)	1.063^b^	0.303
High blood pressure or diabetes	44(19.56%)	20(23.00%)	24(17.40%)		
**Whether to remove the breast**					
Yes	154(68.44%)	62(71.30%)	92(66.70%)	0.522^b^	0.470
No	71(31.56%)	25(28.70%)	46(33.30%)		
**Chemotherapy cycles**					
< 8 cycles	51(22.67%)	24(27.60%)	27(19.60%)	1.958^b^	0.162
≥ 8 cycles	174(77.33%)	63(72.40%)	111(80.40%)		
**Neoadjuvant chemotherapy**					
Yes	86(38.22%)	35(40.20%)	51(37.00%)	0.242^b^	0.623
No	139(61.78%)	52(59.80%)	87(63.00%)		
**Radiotherapy**					
Yes	91(40.44%)	34(39.10%)	57(41.30%)	0.11^b^	0.741
No	134(59.56%)	53(60.90%)	81(58.70%)		
**Endocrine therapy**					
Yes	117(52.00%)	42(48.30%)	75(54.30%)	0.788^b^	0.375
No	108(48.00%)	45(51.70%)	63(45.70%)		
**Targeted therapy**					
Yes	52(23.11%)	17(19.50%)	35(25.40%)	1.018^b^	0.313
No	173(76.89%)	70(80.50%)	103(74.60%)		
**Ovarian inhibitor therapy**					
Yes	26(11.56%)	12(13.80%)	14(10.10%)	0.695^b^	0.405
No	199(88.44%)	75(86.20%)	124(89.90%)		
**Disease stage**					
I	63(28.00%)	20(23.00%)	43(31.20%)	2.778^b^	0.249
II	104(46.22%)	46(52.90%)	58(42.00%)		
III and IV	58(25.78%)	21(24.10%)	37(26.80%)		
**Age(years)**	47.52 ± 7.15	47.61 ± 7.83	47.46 ± 6.71	0.48 ^a^	0.882
**Marriageable age**	20.00(26.00–31.00)	26.00(23.03-27.00)	25.50(23.41–25.96)	-0.633^c^	0.527
**BMI**	21.48(23.11–25.49)	21.48(23.53–25.78)	22.93(22.98–24.03)	-0.38^c^	0.704
**Libido**	2.40(3.60–3.60)	2.40(2.40–3.60)	3.60(3.00-3.60)	14.112^c^	<0.001
**Sexual arousal**	3.90(4.50–4.80)	3.90(3.60–4.80)	4.50(4.20–4.80)	9.807^c^	0.002
**Vaginal lubrication**	4.20(4.80–5.40)	4.50(3.90–4.80)	4.80(4.80–5.48)	14.399^c^	<0.001
**Climax**	3.60(4.40–4.80)	3.60(3.20–4.40)	4.40(3.60–4.80)	10.41^c^	0.001
**Sexual satisfaction**	4.40(4.80–5.20)	4.80(4.40–4.80)	4.80(4.80–5.60)	10.038^c^	0.002
**Sexual pain**	4.60(5.20-6.00)	4.80(4.00-5.20)	5.20(4.80-6.00)	10.486 ^c^	0.001

Note: In statistics, “a” refers to *t* test, “b” refers to *χ*^*2*^ test, and “c” refers to rank sum test

**Table 2:** Statistical description of sexual activity of patients in the two groups at each assessment point(*n* = 225)

point-in-time	T_0_	T_1_	T_2_	T_3_	T_4_
sexual activity	225(100%)	88(39.1%)	118(52.4%)	111(51.4%)	89(63.1%)

**Table 3:** Trajectory model fitting results

Indicators	Optimal number of tracks	BIC value	App value	
			Subgroup 1	Subgroup 2
sexual activity	2	-522.12	0.933(0.503–0.986)	0.992(0.903–0.999)

**Table 4:** Multivariate binary logistic regression analysis was used to test the factors affecting the trajectory grouping of breast cancer patients with sexually active

Variables	β	SE	Wald	*P*	OR	95%CI
Age	0.085	0.026	10.684	0.001	1.089	1.035 ∼ 1.145
Libido	0.774	0.247	9.851	0.002	2.168	1.337 ∼ 3.515
Vagina lubrication	1.254	0.286	19.208	<0.001	3.503	2.000 ∼ 6.137
Constant	-12.030	2.337	26.501	<0.001	<0.001	

### Comparisons of demographic characteristics

An overview of the demographic characteristics was provide in Table 1. The mean age of the 225 breast cancer patients was 47.52 ± 7.15, the youngest was 28 years old and the oldest was 69 years old, 66.2% of the patients were in non-menopausal status, the cancer stage was mostly stage I and II (74.2%), 91.1% of the patients received surgery and 99.1% received chemotherapy.

### Sexual activity

Statistical description of the sexual activity of the two groups of patients shows the time points of the sexually active ratio(see Table 2). At T_0_, 225 patients (100%) had sex in the past month. This percentage greatly decreased to 88 patients (39.1%) at T_1_ and then increased gradually thereafter to 118 patients (52.4%) at T_2_, 111 patients (51.4%) at T_3_, and 89 patients (63.1%) at T_4_.

### Trajectory analysis

The BTM showed that the optimal number of sexually active trajectories for breast cancer patients was 2 groups, yielding a BIC value of -522.12 and Avepp values all greater than 0.9 (see Table 3). We identified 2 trajectories of sexual activity status (see Fig. 2). Subgroup 1 accounted for 36.6% of the total number of patients. The sexual activity of patients in this group decreased significantly from T_1_ to T_3_, and the activity level was close to zero and increased to approximately 40% at T_4_, which was defined as the “low activity group”. Subgroup 2 accounted for 63.4% of the total number of patients, and the sexual activity of this group was the lowest point at T_1_ and remained high at T_2_-T_4_, with an activity level of approximately 80%, which was defined as the “high activity group”.

### Multifactorial analysis of sexually active trajectory grouping in breast cancer patients

The sexually active trajectory grouping was used as the dependent variable, and the demographic characteristics of breast cancer patients (age, residence, marriage, occupation, income, education, type of health insurance, menstrual status), disease and treatment information (BMI, underlying disease, surgical modality, chemotherapy cycle, neoadjuvant chemotherapy, radiotherapy, endocrine therapy, targeted therapy, ovarian suppressant, pathological stage), and baseline levels of patient sexual function (libido, sexual arousal, vaginal lubrication, orgasm, sexual satisfaction, painful intercourse) as independent variables to analyze the factors influencing different trajectory types.

### Univariate analysis of sexually active trajectory groupings

Univariate analysis comparing the general demographic data, clinical data and patients’ pre-treatment sexual function levels between the low and high active groups showed statistically significant differences in sexual desire, arousal, vaginal lubrication, orgasm, sexual satisfaction, and painful intercourse between the two groups at baseline(Table 1).

### Multifactorial analysis of sexually active trajectory grouping

Even if the variables have a significance level of *P* < 0.1 in the single factor analysis, those variables that may have a significant impact are included as independent variables in the binary logistic regression equation. The influence of a multivariate binary logistic regression model on the grouping of patients’ sexual activity trajectory, as shown in Table 4, is examined. The multivariate binary logistic regression analysis revealed statistically significant and positive correlations between sexual activity and age (*β* = 0.085, OR = 1.089, 95%CI 1.035 ∼ 1.145, *P* = 0.001),libido(*β* = 0.774, OR = 2.168, 95%CI 1.337 ∼ 3.515, *P* = 0.002)vaginal lubrication(*β* = 1.254, OR = 33.503, 95%CI 2.000 ∼ 6.137, *P*<0.001). The results showed that the older the patients, the higher the libido and vaginal lubrication scores, the higher the level of sexual activity, and the differences were statistically significant (see Table 4).

## Discussion

The aim of this study was to investigate the overall activity status of breast cancer patients during treatment after the first be diagnosed, with the objective of elucidating the trajectory phenomenon of life. As the treatment progressed, the proportion of sexual activity showed a tendency to decrease first and then increased after diagnosis. The sexual activity of breast cancer patients in this study at each follow-up time point was T_1_ 39.1%, T_2_ 52.4%, T_3_ 51.4%, and T_4_ 63.1%, which was closer to the results of the German scholar Farthmann’s study [49]. However, the level of sexually active ratio varies at different stages of the disease.

All patients became sexually active before diagnosis, but there was some time of sexual suspension after diagnosis. Therefore, the accurate diagnosis of diseases plays a pivotal role in influencing patients’ sexual behavior. After the patient was diagnosed, the proportion of patients with sexual activity in DU Hua’s study was higher than 5.02% [26] and the 20.83% reported by Cavalheiro et al. [50], reaching 39.1%.This result suggests that with social development, breast cancer patients place a higher value on sexuality after the disease and may also be related to the difference in treatment status that the patients were in at the time of the survey.

The different tendency of the sexual activity during treatment may be due to different criteria for evaluating and defining sexual activity in breast cancer patients. Chinese researcher Yuan Xiaoling distinguished active from inactive by whether patients had sex in the past 1 month [44]. American scholar Avis defined sexually active by asking patients whether they were sexually active in the past 1 month [27], and Korean scholar Maria Lee identified patients who had sex more than 1 time per month as type active [32]. Although there are some differences in the current definition of sexually active in different countries, for this special group, we believe that patients who can have sexual intercourse 1 and more times in the past 1 month can be considered sexually active. Approximately half of the patients in this study suspended their sexual life during treatment continuation, which was consistent with the Farthmann study (55.6%). In the present results, the level of sexual activity of patients in the early recovery stage (9 months after diagnosis and approximately 3 months after finishing antitumor treatment) was 63.1%, which was higher than that in the study by Italian scholar Biglia et al. [51] (34.3%∼37.1%) but lower than that reported by Belgian scholar Aerts et al. [52] (64%∼71%). Forty of these patients (17.8%) completely suspended sex throughout the follow-up period, which was lower than the 29.29% in the DU Hua study [26]. Our findings indicated that the overall activity exhibited a higher level in patients diagnosed with breast cancer, but the level of sexual activity in breast cancer patients needs further improvement. The overall trends in sexual behavior may be attributed to variations in the treatment status of patients during the survey period, highlighting the importance of early intervention and recovery to enhance and restore patients’ sexual well-being in tandem with disease progression. Since the maximum follow-up period of this study was 9 months after diagnosis, the best level and time point of sexual activity recovery in patients could not be explored, and further studies are needed [27, 32].

The sexual activity trajectories of patients exhibited significant heterogeneity and pronounced polarization. This research also presents a significant contribution to the field of innovation, as it delves into unexplored territory by categorizing sexual activity levels and examining their trajectory. The trajectory model of sexual activity development showed two trends in the sexual activity status of breast cancer patients during the treatment phase: the “high active group” accounted for 62.3% of the patients, who were active at approximately 50% at T_1_ and recovered to a high level of approximately 80% at T_2_∼T_4_; the “low active group. The percentage of the “low active group” was 37.7%, and the sexual activity was very low throughout the follow-up period, close to 0, and recovered only approximately 40% at T_4_. The results of this study showed that there is significant heterogeneity in the sexual activity status of breast cancer patients after the disease. Sexually active patients with high and low activity should be given different intervention protocols. Patients in the high active group were sexually active up to 50% at the T_1_ stage, suggesting that clinical interventions on sexual function of breast cancer patients can be carried out at an early stage to help patients establish correct cognition of sexual life, better restart their sexual life and improve the level of sexual activity. For patients with high sexual activity levels, we can focus on sexual life quality improvement to reduce the influence of disease and treatment factors on sexual life quality. For patients with a low level of sexual activity, the focus should be on understanding the reasons why the patients stopped having sex, and the intervention should focus on giving the patients the will and confidence to restart their sexual life and further improve the quality of sexual life on this basis.

The trajectory model of sexual activity development provide a rich and valuable information for a deeper understanding of the sexual function status of breast cancer patients. The results of the sexually active group reached about two times the inactive group, indicating that the effect of cancer treatment on the sexual activity level of most patients was limited, and patients still maintained a high level of sexual needs. We need to be confident in the recovery of sexual life of breast cancer patients. The ubiquity of health literacy in China, it helps patients to adapt and recover from the disease to meet needs beyond disease and treatment [52, 53]. The clinical features of the disease need to be further explored as to why this change occurs. We clarify the different levels of sexual activity and related factors and improve the level of individualized intervention.

The trajectory analysis results in the categorization of individuals into high and low activity groups during a specific time period, aiming to investigate the influential factors. This has significant implications for identifying key determinants that require attention among patients at different time intervals. In this study, patients’ basic demographic information, clinical treatment, disease information, and pretreatment sexual function level [54] were included in the analysis of factors influencing the trajectory of sexual activity to provide a basis for predicting changes in the development of sexual activity after patient diagnosis on the one hand and to explore whether the baseline level of patients’ sexual function at baseline has an impact on the trajectory of sexual activity from various dimensions of patients’ sexual function at baseline, with a view to providing future interventions. The results of multivariate binary logistic regression analysis suggested that the older the patients, the higher the libido and vaginal lubrication scores, the higher the level of sexual activity. This demonstrates that neither demographic nor clinical information other than age was an influential factor in the trajectory of sexual activity, suggesting that the age of breast cancer patients exhibits a stronger correlation with sexual activity and sexual function. Patients’ pretreatment sexual desire and vaginal lubrication were independent influences on the trajectory of sexual activity. Patients with good basal sexual desire and vaginal lubrication were more inclined to have a high level of sexual activity during treatment, independent of clinical information about the disease. The results are not entirely consistent with the findings of previous studies. A Korean longitudinal study that followed patients from diagnosis to six months after the end of treatment found that receiving chemotherapy, thyroid dysfunction, and depression were risk factors for sexual inactivity [55, 56]. Yuan [44] showed through a cross-sectional survey that older age, low economic level, health insurance for NPS and those without health insurance, and receiving chemotherapy were influential factors for low sexual activity in breast cancer patients.

Previous studies examining influencing factors have primarily focused on cross-sectional changes and have indicated a correlation between sexual activity and age as well as other variables. This prospective study revealed that individuals aged over 50 engage in more sexual activity compared to younger individuals, which contradicts conventional beliefs [54]. That said, patients in their 50s were found to maintain higher levels of sexual activity following diagnosis. In contrast, younger women exhibited lower levels of sexual activity, engaging in intercourse less frequently or suspending it altogether. The reason for this age disparity in sexual activity during the specified time period is attributed to greater physiological changes related to sexual function before and after treatment among younger breast cancer patients compared to older ones [57], making sexual adaptation and recovery more challenging for the latter group. Research has demonstrated that oncology treatments often have a more detrimental impact on younger women who are prone to experiencing menopausal symptoms due to ovarian failure and are affected by post-surgical alterations in body image [58, 59]. These factors can be psychologically and physically devastating, resulting in decreased libido, reduced sexual activity, and an overall decline in quality of life [60, 61]. On the other hand, older patients—particularly those who have undergone menopause—have already adapted to the postmenopausal physiological state and do not experience obvious endocrine-related side effects caused by drug therapy. Therefore, it is crucial that we prioritize interventions targeting the sexually active vulnerable group of young breast cancer patients with a focus on improving their sexual activity and quality of life. This approach will play a significant role in enhancing overall breast cancer health outcomes in the future. Another important reason for this difference is that this study breaks the traditional step of assessing sexual function in breast cancer patients by first knowing whether the patient is sexually active and then assessing sexual function. In addition, the trajectory model of sexual activity during treatment of breast cancer patients in this study is based on 5 follow-up data points, and the focus of our study is on the trajectory change trend of sexual activity in a specific period of time, rather than a single point in time, providing a comprehensive and longitudinal perspective of the stage development of sexual activity. This methodology allows medical professionals to gain deeper insights into the characteristics and patterns of sexual activity within this population, thereby enhancing the persuasiveness and credibility of our findings.

Few studies have focused on the impact of patients’ underlying sexual function status on the quality of sexual life after the disease. According to the DSM 5th edition published by APA in 2013, sexual desire disorders are mainly divided into hypoactive and aversive sexuality, when patients reduce, avoid or even avert the occurrence of sexual life, resulting in a reduction in the frequency of sexual life or even its suspension [62]. Thus, sexual desire is directly related to sexual activity. We found that patients with good levels of basal libido and vaginal lubrication may better cope with decreased libido and reduced vaginal lubrication brought about by treatment. It directly affects their adaptation and recovery of their sexual function status after the disease. Most of the patients included in our study were around menopause. More of them already had reduced vaginal discharge before treatment, which was further aggravated by a series of antitumor treatments, especially chemotherapy and endocrine therapy, with damage to the endocrine system and difficulties in vaginal lubrication, the most obvious manifestation of which is the reduction of vaginal discharge as a structural change of the reproductive organs. Directly dryness and difficulties in sexual intercourse the most apparent manifestation is the reduction of vaginal secretions, a structural change in the genital organs, which directly leads to dryness and difficulty in sexual intercourse, thus reducing or even refusing sex [56, 63].

### Limitations

There are 3 limitations of this study. First, although the study population was obtained from tertiary care hospitals specializing in oncology in Southwest China, the study population has some limitations because it is a single-center study. Second, although the study subjects were followed up for 9 months, the observation period was relatively short. Thirdly, owing to the absence of a dedicated scale for measuring sexual activity, various dimensions of the FSFI scale were employed for preliminary investigation into clinical assessment of sexual activity in order to comprehend the trajectory and influencing factors associated with patients’ sexual functioning.

### Future direction

Since sexual function changes over time, further studies with larger sample sizes and multicenter studies are needed to observe sexual function between low- and high-activity groups. Considering the physiological and psychological problems caused by surgery and endocrine therapy for young breast cancer patients, which can lead to loss of libido and sexual dysfunction, the sexual function of young female breast cancer patients is a key concern.

## Conclusions

Breast cancer patients experience a decrease in sexual activity upon diagnosis, and throughout treatment, there is fluctuation between high and low levels of sexual activity. These variations are influenced by factors such as age, pre-diagnosis level of sexual desire, and vaginal lubrication. In clinical practice, it is crucial to prioritize health education on sexual function among young patients.

### Electronic supplementary material

Below is the link to the electronic supplementary material.

Supplementary Material 1

## Data Availability

The data supporting this study’s findings are available from the corresponding author upon reasonable request.
