# Supplementary material for: A longitudinal study of sexual activity and influencing factors in breast cancer patients during treatment in the Southwest of China: a trajectory analysis model
Source: BMC Womens Health. 2024 Jun 18;24:352. doi: 10.1186/s12905-024-03150-8 (PMC11184704; doi:10.1186/s12905-024-03150-8)
Supplement: Supplementary file 1 — Supplementary Material 1 [file 12905_2024_3150_MOESM1_ESM.docx]

# What this paper adds

- This study investigated the overall changes in patients' sexual activity and the time points at which changes occurred from the time of illness, during treatment, early recovery by longitudinally investigating patients.
- This study breaks the traditional steps of sexual function assessment in breast cancer patients, based on understanding whether patients are sexually active or not, and then conducting sexual function assessment.
- This study prospectively found that those aged >50 years were more sexually active than those younger, implying that younger breast cancer patients are a priority population for our sexual life intervention.
